# Pharyngeal Pumping and Tissue-Specific Transgenic P-Glycoprotein Expression Influence Macrocyclic Lactone Susceptibility in *Caenorhabditis elegans*

**DOI:** 10.3390/ph14020153

**Published:** 2021-02-13

**Authors:** Alexander P. Gerhard, Jürgen Krücken, Cedric Neveu, Claude L. Charvet, Abdallah Harmache, Georg von Samson-Himmelstjerna

**Affiliations:** 1 Institute for Parasitology and Tropical Veterinary Medicine, Freie Universität Berlin, 14163 Berlin, Germany; alexander.gerhard@fu-berlin.de (A.P.G.); juergen.kruecken@fu-berlin.de (J.K.); 2 INRAE, Université de Tours, ISP, F-37380 Nouzilly, France; cedric.neveu@inrae.fr (C.N.); claude.charvet@inrae.fr (C.L.C.); abdallah.harmache@inrae.fr (A.H.)

**Keywords:** macrocyclic lactones, nematode, parasitology, P-glycoprotein, drug resistance, macrocyclic lactones, *Caenorhabditis elegans*

## Abstract

Macrocyclic lactones (MLs) are widely used drugs to treat and prevent parasitic nematode infections. In many nematode species including a major pathogen of foals, *Parascaris univalens*, resistance against MLs is widespread, but the underlying resistance mechanisms and ML penetration routes into nematodes remain unknown. Here, we examined how the *P*-glycoprotein efflux pumps, candidate genes for ML resistance, can modulate drug susceptibility and investigated the role of active drug ingestion for ML susceptibility in the model nematode *Caenorhabditis elegans*. Wildtype or transgenic worms, modified to overexpress *P. univalens* PGP-9 (*Pun*-PGP-9) at the intestine or epidermis, were incubated with ivermectin or moxidectin in the presence (bacteria or serotonin) or absence (no specific stimulus) of pharyngeal pumping (PP). Active drug ingestion by PP was identified as an important factor for ivermectin susceptibility, while moxidectin susceptibility was only moderately affected. Intestinal *Pun*-PGP-9 expression elicited a protective effect against ivermectin and moxidectin only in the presence of PP stimulation. Conversely, epidermal *Pun*-PGP-9 expression protected against moxidectin regardless of PP and against ivermectin only in the absence of active drug ingestion. Our results demonstrate the role of active drug ingestion by nematodes for susceptibility and provide functional evidence for the contribution of *P*-glycoproteins to ML resistance in a tissue-specific manner.

## 1. Introduction

The treatment of parasitic nematode infections in animals and humans relies on chemotherapy, and macrocyclic lactones (MLs) represent the most widely used drug class in veterinary medicine due to their high efficacy, low toxicity, and broad spectrum of target parasites [1]. However, drug resistance to MLs is now widespread in parasitic nematodes of ruminants [2], horses [3], companion animals [4,5], and humans [6]. With over 1.5 billion infected humans [7] and essentially all domestic animals exposed to nematode infections, the ongoing development and spread of ML drug resistance is an obstacle to maintaining health standards. In the pathogenic equine parasite *Parascaris univalens*, resistance against MLs is widespread, which poses a health threat to young animals worldwide [8].

In *Caenorhabditis elegans* and in parasitic nematodes, the primary pharmacological targets of MLs are the glutamate-gated chloride channels (GluCls) [9,10]. The irreversible binding of MLs to GluCls leads to a hyperpolarisation of the respective neurons that control locomotion, pharyngeal pumping, and egg laying, resulting in flaccid paralysis of the muscles and pharynx [9,10]. However, the underlying resistance mechanisms are insufficiently understood.

The ATP-binding-cassette subfamily B member 1 (ABCB1) transporter genes, more commonly referred to as P-glycoproteins (Pgps), were among the first candidate genes for ML resistance in parasitic nematodes [11,12] and in *C. elegans* [13,14]. These xenobiotic efflux pumps are conserved in eukaryotes and have a broad lipophilic substrate range [15]. Their role in ML resistance has been inferred from observed differences of individual Pgp expression in association with drug exposure [16] and findings of constitutively high expression levels in resistant compared to susceptible isolates [11]. Recently, next-generation sequencing (NGS) approaches allowed the identification of genetic signatures of selection in close proximity to Pgp loci in resistant and introgressed populations [17,18,19]. In addition, several functional studies in *C. elegans* have provided evidence for an involvement in the ML resistance phenotype. For example, individual Pgp deletion strains, and silencing or inhibition of Pgps increased ivermectin (IVM) susceptibility [13,14]. However, our understanding of how Pgps contribute to ML resistance functionally remains superficial and fragmented. In contrast to mammals, which only possess one (human) to three (rodents) Pgps, the nematode Pgp gene family comprises a diverse repertoire that differs between nematodes species [20], e.g., 15 Pgps in *C. elegans* [21] and 10 Pgps in *Haemonchus contortus* [22]. More recently, the Pgp repertoire of *P. univalens* was completely deciphered, revealing 10 different Pgp genes with particularly strong expression levels for *Pun-pgp-11.1, Pun-pgp-16.2*, and *Pun-pgp-9* [23]. Across different species, the *pgp-9* gene lineage has been repeatedly associated with ML resistance in several nematode species such as cyathostomins [19,24], *Teladorsagia circumcincta* [18,25], and *H. contortus* [26] and is regarded as one of the most relevant candidates for a contribution to ML resistance. While Pgps are expressed in most tissues, expression is most prominent in the intestine in different nematode species, including *P. univalens* and *C. elegans* [23,27,28,29]. In addition, the epidermis (= hypodermis) also exhibits moderate Pgp expression levels in *P. univalens* [27] and *C. elegans* [23], but the tissue-specific function of these genes is unclear.

Many marketed ML derivatives have been shown to exhibit differing pharmacokinetics, efficacies, and chemical properties [1]. Generally, MLs are classified into two groups, the avermectines (including IVM) and the milbemycins (including moxidectin, MOX), the latter lacking a polysaccharide side chain at C13 of the lactone ring. This chemical disparity has been suggested to lead to the differences in the affinity of nematode Pgps for different ML derivatives [24,30]. In mammals, the main route of ML elimination is the intestine, and here, Pgps have been shown to play an important role [31]. In contrast, the uptake and elimination routes of MLs in nematodes and the role of Pgps in these processes remain to be elucidated. Mammalian Pgps also reduce the permeation of MLs through the blood–brain barrier and Pgp (i.e., *ABCB1* or *mdr-1*) deficiency causes acute neurotoxicity upon IVM treatment [32]. In this regard, the capacity of MDR-1 to restrict effective crossing of MOX and IVM over the barrier varies between IVM and MOX [33].

In the present study, we investigated whether the tissue-specific Pgp expression patterns in nematodes are relevant for modulation of ML susceptibility. Based on their role in mammals, we hypothesised that intestinal and epidermal Pgp expression in nematodes can reduce ML permeability of these tissues. Our objective was to examine the effect of tissue-specific Pgp overexpression at the intestine and at the epidermis on IVM and MOX susceptibility using transgenic overexpression of the ML resistance candidate gene *Pun*-PGP-9 in *C. elegans*. Additionally, we took advantage of *C. elegans* behaviour to induce active drug ingestion by pharyngeal pumping (PP) in the presence of bacteria [34] or through chemical stimulation of PP by 5-hydroxytryptamine (5-HT = serotonin) [35], while in turn mostly limiting exposure to the cuticle–epidermis barrier in the absence of an appropriate PP stimulus [36,37]. Our results show that active drug ingestion increases susceptibility to IVM considerably and to MOX only moderately and indicate that both derivatives are taken up by *C. elegans* via the intestine and cuticle–epidermis. In both tissues, Pgps have a protective function by reducing the worms’ susceptibility to IVM and MOX.

## 2. Results

### 2.1. Tissue-Specific Expression Patterns of *Pun-PGP-9* in a *Cel-pgp-9* Mutant Strain

To address the function of *Pun-pgp-9* using *C. elegans*, the *Cel-pgp-9 (tm830)* null allele was used to generate transgenic strains carrying extrachromosomal arrays driving tissue-specific overexpression of *Pun-PGP-9*. Two lines, henceforth referred to as intestine-Pgp-9 line 1 (*IntPgp-9Ex1*) and line 2 (*IntPgp-9Ex2*), were generated with *Pun*-PGP-9 expression driven by the intestine-specific *gut esterase 1* promotor (*ges-1p*) [38] and pharyngeal green fluorescence protein (GFP) expression. Another line, henceforth addressed as epidermis-Pgp-9, was generated with *Pun*-PGP-9 expression driven by an epidermis-specific *collagen-19* promotor (*col-19p*) [39] and pharyngeal GFP expression (*EpiPgp-9Ex1).* Finally, a line serving as the control strain expressing only GFP in the pharynx (*myo-2p*::*gfp*) (*CtrlEx1*) was generated.

**Figure 1:**
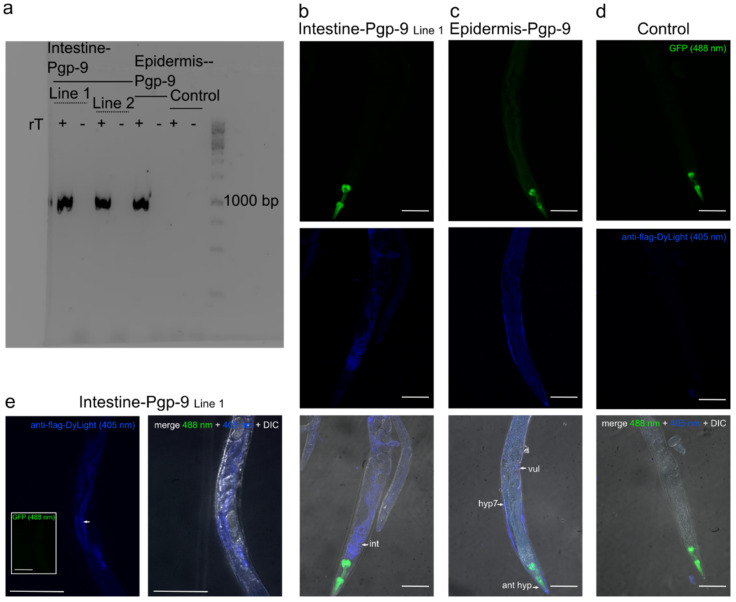
Tissue-specific expression of *Pun*-PGP-9 in *Cel-pgp-9* loss-of-function strain, strain tm830. Verification of transcription and tissue-specific expression. (**a**) Visible bands for all reverse-transcriptase (RT) PCRs on cDNA templates made from whole-worm total RNA, and no bands for no RT controls and the control strain. (**b**–**e**) Confocal microscope images of tissue-specific expression patterns of fixed, freeze cracked, and immunofluorescence-stained transgenic adult *Caenorhabditis elegans* (blue lookup table (LUT), 405 nM excitation), pharyngeal GFP expression (green LUT, 488 nm excitation) and merged images (DIC +405 nM + 488 nM). Primary antibodies target the FLAG-tag fused to *Pun*-PGP-9 and the secondary antibody is conjugated with DyLight405. Images were acquired with a confocal Eclipse Ti-U inverted research microscope and processed and merged with ImageJ [40]. All scalebars are 100 µm. (**b**) Immunostaining in the intestine-Pgp-9 line 1 (white arrows indicate the intestine) and GFP expression at the pharynx. (**c**) Immunostaining of the epidermis-Pgp-9 strain (white arrows indicate the epidermal syncytium) and pharyngeal GFP expression. (**d**) Immunostaining in the control strain and pharyngeal GFP expression. (**e**) Higher magnification of the intestine-Pgp-9 strain (white arrow indicates the apical membrane), *Pun*-PGP-9: *Parascaris univalens* P-glycoprotein-9, GFP: green fluorescence protein, DIC: differential interference contrast, int: intestine, vul: vulva, hyp7: hyp7 syncytium, N2∆*Cel*Pgp*-9* is tm830 (NBRP) [*Cel-pgp-9(-)*]; Transgenic strains genotypes: Epidermis-Pgp-9 *EpiPgp-9Ex1* [*Cel-pgp-9(-); Cel-col-19p::Pun-pgp-9::FLAG::Cel-unc-54_3′-UTR; Cel-myo-2p::gfp:: Cel-unc-54_3′UTR*]; Intestine-Pgp-9 Line 1 *IntPgp-9Ex1* [*Cel-pgp-9(-); Cel-ges-1p::Pun-pgp-9::FLAG::Celunc-54_3′-UTR; Cel-myo-2p::gfp::Cel-unc-54_3′UTR*]; Control strain *CtrlEx1* [*Cel-pgp-9(-); Cel-myo-2p::gfp:: Cel-unc-54_3′-UTR*].

Transcription of *Pun-pgp-9* was confirmed by RT-PCR for all *Pun*-PGP-9 transgenic strains by amplifying a 1043 bp PCR product, while no PCR product was amplified in the control strain (Figure 1a). Protein localisation in the epidermis and the intestine were confirmed by the immunofluorescence detection of a FLAG-tag fused to the NH_2_-end of *Pun*-PGP-9 and visualised using a secondary DyLight405 antibody and confocal microscopy (Figure 1b–e). *Pun*-PGP-9 protein expression in intestine-Pgp-9 line 1 appeared intestinal specific (Figure 1b). At a higher magnification, a strong apical expression was apparent (Figure 1e). Expression in the epidermis-Pgp-9 strain was detectable in the epidermis (Figure 1c). Specific blue fluorescence was not visible in the control strain following staining, while pharyngeal GFP expression was visible, indicating that the antibody staining was specific to the FLAG-tagged *Pun*-PGP-9 (Figure 1d).

### 2.2. Motility Assays in Transgenic and Wildtype *Caenorhabditis elegans*

All mentioned half-maximal effective concentration (EC_50_) values, fold changes, and other parameters calculated from the non-linear regression models as well as p-values from statistical significance tests are given in Supplementary Table S1 for IVM and Supplementary Table S2 for MOX and are not specifically referenced in the text.

**Figure 2:**
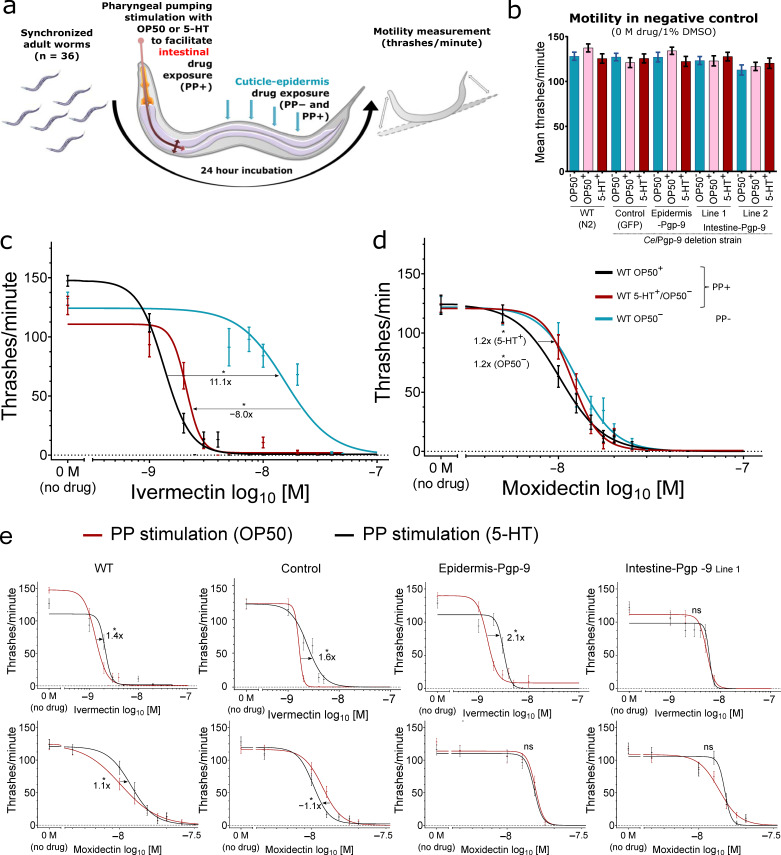
Pharyngeal pumping increases ivermectin and moxidectin susceptibility. Effect of pharyngeal pumping (PP) stimulation by OP50 food bacteria or serotonin in *Caenorhabditis elegans* on ivermectin and moxidectin susceptibility. (**a**) Schematic illustration of the experimental setup with active ingestion and intestine drug exposure by PP stimulation. (**b**) Mean thrashes/minute ± standard error of the mean (SEM) in the negative control (no drug, 1% DMSO) between strains and conditions. Each strain/condition combination was compared to WT OP50^−^ with a Kruskal–Wallis test with Dunn’s post hoc, and *p* > 0.05 was considered not significant. (**c**,**d**) Ivermectin and moxidectin concentration–response curves calculated with GraphPad v8.3.0 based on thrashes/minute in the WT strain with n = 36 per concentration spread equally on three separate days. PP stimulation by OP50 bacteria (OP50^+^) (black), 4 mM 5-HT (red), or in the absence of a PP stimulus (OP50^−^) (blue). Significant differences between half-maximal effective concentration (EC_50_) were calculated using the extra-sum-of-squares-F test and Bonferroni correction. (**e**) Comparison of the effect of PP stimulation by OP50 *Escherichia coli* food bacteria (red) or 5-HT (black) in different transgenic and wildtype strains. (**c**–**e**) All calculated four parameter non-linear regression models and SEM at each concentration correspond to Supplementary Tables S1 and S2. Prior to the calculation, the no-drug negative control was set to 0.1 nM and all concentrations were log_10_ transformed. On the x-axis, the negative control was visualised as “0 M (no drug)” and separated by a break in the axis. *P*-values < 0.05 were considered significant and are indicated with an asterisk, while corresponding fold-changes are indicated with arrows. 5-HT: serotonin/5-hydroxytryptamine; WT: *C. elegans* N2 Bristol; Control strain genotype *CtrlEx1* [*Cel-pgp-9(-); Cel-myo-2p::gfp:: Cel-unc-54_3′-UTR*].

Motility assays were conducted as described elsewhere [41] with modifications for the stimulation of PP as schematically summarised in Figure 2a. Prior to drug incubations, induction of PP by feeding OP50 or 5-HT treatment or the lack of PP in the absence of a stimulus was confirmed visually under a stereo microscope. By PP stimulation, MLs dissolved in DMSO within the incubation medium were actively ingested. After an 18–24 h incubation, the motility of worms incubated without drugs in 1% DMSO (negative, vehicle control) was tested; neither transgene expression nor induction of pharyngeal pumping by 5-HT or OP50 significantly influenced motility (Kruskal–Wallis test with Dunn’s post hoc test) compared to the wildtype (WT, untransformed N2 Bristol strain worms)/OP50^−^ condition (no PP stimulation) (Figure 2b).

#### 2.2.1. The Effect of Active Drug Ingestion on Ivermectin and Moxidectin Susceptibility

Strikingly, the EC_50_ for the WT in the OP50^+^ condition was 11.1-fold lower than in the absence of OP50 bacteria (Figure 2c). In order to evaluate whether this effect is the result of potentially confounding factors such as IVM metabolisation by the OP50 bacteria or starvation, OP50 were substituted by 5-HT in the incubation medium, thereby eliminating the bacteria and food source but maintaining a stimulus for pharyngeal pumping. This resulted in an 8.0-fold decrease in the EC_50_ compared to OP50^−^ to a level just above that achieved by incubation with OP50 bacteria (Figure 2c). 

For MOX, stimulation of PP by OP50 also resulted in overall lower EC_50_ values than in the absence of PP (OP50^−^), but the effect of PP was considerably smaller than that observed for IVM (Figure 2c,d). 

Comparing PP induction by 5-HT^+^ and OP50^+^, IVM EC_50_ values were higher when stimulated by 5-HT across all strains except intestine-Pgp-9 (all *p* < 0.05, extra-sum-of-squares F test, Bonferroni corrected) while MOX EC_50_ values varied slightly in the control and WT and were not significantly different in both *Pun*-PGP-9 expressing strains (both *p* > 0.05, extra-sum-of-squares F test, Bonferroni corrected) (Figure 2e).

#### 2.2.2. *Cel-pgp-9* Loss-of-Function Strain Susceptibility Phenotype in Adult Stage

The control strain, which is the *Cel-pgp-9* null mutant (*CtrlEx1 [Cel-pgp-9(-); Cel-myo-2p::gfp:: Cel-unc-54_3′-UTR*]), did not exhibit increased susceptibility to IVM or MOX (Supplementary Figure S1a,b). Serving as a control for extrachromosomal array transgene expression (pharyngeal GFP expression), the calculated EC_50_ values in the control strain matched those of the WT for IVM. Likewise, the control strain’s response to MOX was similar to that of the WT strain despite minor variations (Supplementary Figure S1c,d). The control strain was used as a reference for both the *Pun*-PGP-9 overexpression strains, epidermis-Pgp-9 and intestine-Pgp-9, as it exhibits the same genetic background and transgenic extra-chromosomal expression.

#### 2.2.3. The Effect of Epidermal Pun-PGP-9 Expression on Ivermectin and Moxidectin Susceptibility

Epidermal *Pun*-PGP-9 expression reduced susceptibility to IVM only in the absence of PP stimulation but to MOX regardless of PP. 

**Figure 3:**
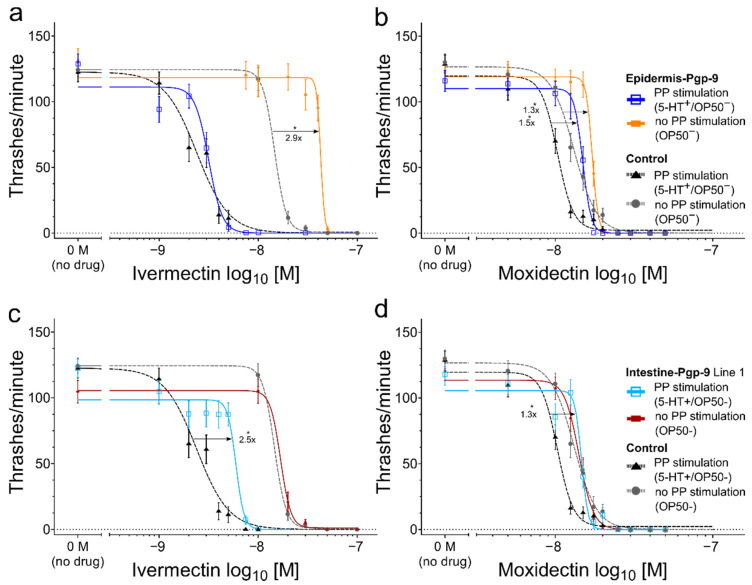
Modulation of ivermectin and moxidectin susceptibility in *C. elegans* by transgenic tissue-specific *Pun*-PGP-9 expression. (**a**–**d**) Concentration–response curves corresponding to Tables S1 and S2 for ivermectin (**a**/**c**) and moxidectin (**b**/**d**) in the absence of a PP stimulus (OP50^−^) or PP stimulation by serotonin (5-HT^+^/OP50^-^) in liquid S-medium. (**a**–**d**) show the control strain’s response for 5-HT^+^ (black dashed line with triangles) or OP50^−^ (grey dashed line with circles), (**a**,**b**) show the epidermis-Pgp-9 strain for 5-HT^+^ (dark blue with open squares) or OP50^−^ (orange with rectangles) and (**c**,**d**) show the intestine-Pgp-9 line 1 for 5-HT^+^ (light blue with open squares) or OP50^−^ (red with rectangles). All concentration–response curves are based on four-parameter non-linear regression models calculated from the motility response (thrashes/minute) of 36 synchronised 1-day adults per concentration, and error bars represent standard error of the mean. Prior to calculation, concentrations were log_10_ transformed, and the no-drug negative control was set to 0.1 nM. On the x-axis, the negative control was visualised as “0 M (no drug)” and separated by a break in the axis. Significant differences in EC_50_ values were compared using the extra-sum-of-squares-F test and Bonferroni correction; *p*-values < 0.05 were considered significant and indicated with an asterisk (*), while corresponding fold-changes are indicated with arrows. *Pun*-PGP-9: *Parascaris univalens* P-glycoprotein-9; N2∆*Cel*Pgp-*9* is tm830 (NBRP) [*Cel-pgp-9(-)*]; Transgenic strains genotypes: Epidermis-Pgp-9 *EpiPgp-9Ex1* [*Cel-pgp-9(-); Cel-col-19p::Pun-pgp-9::FLAG::Cel-unc-54_3′-UTR; Cel-myo-2p::gfp:: Cel-unc-54_3′UTR*]; Intestine-Pgp-9 Line 1 *IntPgp-9Ex1* [*Cel-pgp-9(-); Cel-ges-1p::Pun-pgp-9::FLAG::Celunc-54_3′-UTR; Cel-myo-2p::gfp::Cel-unc-54_3′UTR*]; Control strain *CtrlEx1* [*Cel-pgp-9(-); Cel-myo-2p::gfp:: Cel-unc-54_3′-UTR*].

Concerning IVM susceptibility, the epidermis-Pgp-9 line resembled the control strain in the presence of an OP50 PP stimulus (Figure 3a), but in the absence of a PP stimulus, the EC_50_ increased significantly by 2.9-fold compared to the control strain. Similarly, the MOX EC_50_ in the absence of PP in the epidermis-Pgp-9 strain was also significantly increased (Figure 3d). However, significantly increased MOX EC_50_ values were observed regardless of PP in this strain (Figure 3b). Notably, the differences to the control strain were only moderate, i.e., a 1.3-fold in the absence of PP (OP50^−^) and a 1.3-fold (OP50^+^) or a 1.5-fold (5-HT^+^) increase in the presence of PP stimulation. 

#### 2.2.4. The Effect of Intestinal *Pun*-PGP-9 Expression on Ivermectin and Moxidectin Susceptibility 

Intestinal *Pun*-PGP-9 expression reduced susceptibility to both tested MLs, but this effect was always dependent on active drug ingestion.

Concerning IVM, intestinal *Pun*-PGP-9 expression reduced the susceptibility to IVM in the presence of a PP stimulus compared to the control strain (Figure 3c) in two independent lines (Supplementary Figure S1c,d). In contrast, IVM EC_50_ values for both lines were not significantly different from the control strain when PP was not stimulated (Figure 3c).

MOX EC_50_ values in the two intestine-Pgp-9 lines were also significantly elevated when PP was stimulated by OP50 or 5-HT compared to the control strain (Figure 3d). However, in contrast to IVM, the corresponding fold-changes were considerably smaller (<1.3-fold). In addition, all MOX EC_50_ values for the two lines were overall lower than the MOX EC_50_ values calculated for the epidermis-Pgp-9 line. In the absence of PP stimulation, EC_50_ values did not increase compared to the control strain, which is in line with the observations for IVM.

#### 2.2.5. A Comparison of Moxidectin and Ivermectin Susceptibility in Transgenic and Wildtype Strains

**Figure 4:**
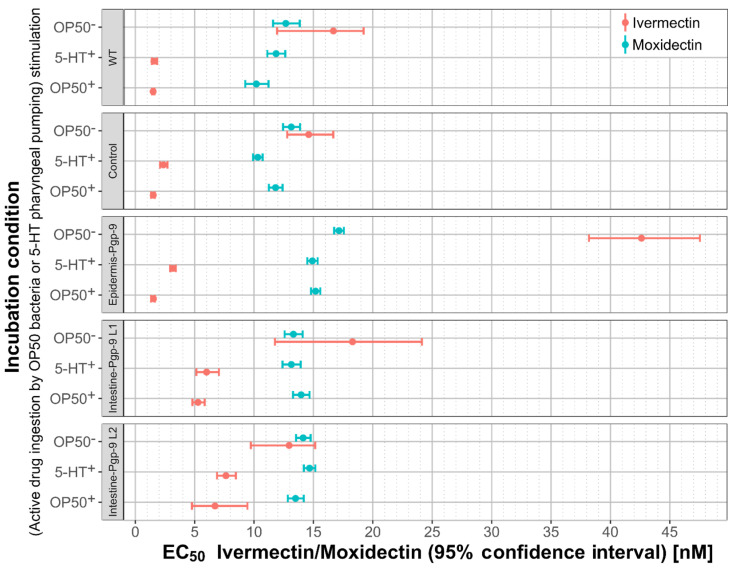
Comparison of moxidectin and ivermectin in wildtype and transgenic *Caenorhabditis elegans* strains. Forrest plot visualising the EC_50_ and corresponding 95% confidence intervals for ivermectin (red) and moxidectin (turquoise) in transgenic and wildtype *Caenorhabditis elegans* strains in the presence of pharyngeal pumping (PP) stimulation by OP50 food bacteria (OP50^+^) or 5-hydroxytryptamine (5-HT^+^) or no PP stimulation (OP50^−^) were visualised using ggplot2 [42] in R v4.0.3 [43]. EC_50_ values were inferred from four-parameter linear regression models calculated from 36 synchronised 1-day adult worms per concentration, strain, and condition using GraphPad v8.3.0. Worms were incubated for 24 h in S-medium containing a concentration series of ivermectin or moxidectin in a final DMSO concentration of 1%. Epidermis-Pgp-9 genotype is *EpiPgp-9Ex1* [*Cel-pgp-9(-); Cel-col-19p::Pun-pgp-9::FLAG::Cel-unc-54_3′-UTR; Cel-myo-2p::gfp:: Cel-unc-54_3′UTR*];. Intestine Pgp-9 Line 1 (L1) and Line 2 (L2) genotype is *IntPgp-9Ex1 or 2* [*Cel-pgp-9(-); Cel-ges-1p::Pun-pgp-9::FLAG::Celunc-54_3′-UTR; Cel-myo-2p::gfp::Cel-unc-54_3′UTR*];. Control strain genotype is *CtrlEx1* [*Cel-pgp-9(-); Cel-myo-2p::gfp::Cel-unc-54_3′-UTR*]; WT is N2 Bristol. Pgp: P-glycoprotein; *Pun*-PGP-9: *Parascaris univalens* P-glycoprotein-9; IVM: ivermectin; MOX: moxidectin; PP: pharyngeal pumping; WT: wildtype.

Compared to IVM, EC_50_ values for MOX were generally higher across strains, when PP was stimulated but lower in the absence of a PP stimulus (Figure 4) as detailed below. For example, MOX EC_50_ values in the WT in the presence of PP stimulation were significantly higher, by 6.7- and 7.6-fold, compared to IVM (Figure 4) for co-incubation with OP50 or 5-HT, respectively. In contrast, when a PP stimulus was not provided, the MOX EC_50_ was not significantly different compared to that of IVM (Figure 4). Across strains, only small fold-changes below 1.2-fold for the MOX EC_50_ values were detected in the presence or absence of PP stimulation, which is in a sharp contrast to those for IVM. As for IVM, a marked EC_50_ increase (up to 11-fold in WT) from PP stimulation to no PP was observed in all strains (Figure 4). Likewise, *Pun*-PGP-9 expression only resulted in low fold changes for MOX EC_50_ values (<1.5-fold) in comparison to considerably higher fold changes (e.g., >3.5-fold in intestine-Pgp-9) for IVM (Figure 4). Despite the marked effect on IVM susceptibility from intestinal *Pun*-PGP-9 overexpression, all EC_50_ values for both intestine-Pgp-9 lines in the presence of PP stimulation remained lower compared to EC_50_ values of any other strain in the absence of a PP stimulus (Figure 4). In contrast, MOX EC_50_ values in the intestine-Pgp-9 strains in the presence and absence of PP were similar (Figure 4).

## 3. Discussion

### 3.1. The Role of Pun-PGP-9 in Ivermectin and Moxidectin Susceptibility

The objective in this study was to elucidate the general functional role of Pgp expression in specific tissues in the context of ML susceptibility. To this end, we chose to exemplarily characterise *Pun*-PGP-9, as it has already been shown to interact with IVM in a yeast model [23]. An important question addressed in this work was whether there are differences in the protective effect of Pgp overexpression between different ML derivatives, by testing IVM as an avermectin and MOX as a member of the milbemycins, which are summarised in Figure 4.

Currently, functional evidence regarding the interaction between specific parasite Pgps and different MLs has mostly been inferred from indirect assays using the above-mentioned yeast model [23,24] or the cell line LLC-PK1 [24,29,30,44,45]. In these model systems, MLs were used as inhibitors for a secondary Pgp substrate with a discernible effect in the model systems to characterise the interaction between a transgenic parasite Pgp and different MLs. These studies have consistently suggested that MOX has a lower affinity to the examined nematode Pgps than IVM. This conclusion is further supported by our findings in *C. elegans* that *Pun*-PGP-9 overexpression induces larger susceptibility shifts for IVM than for MOX, which were observed without the use of a secondary substrate. Whether the low fold-changes for MOX have any biological significance remains to be elucidated [46]. However, *Pun*-PGP-9 overexpression reduced both MOX and IVM susceptibility, which indicates that both MLs are probably *Pun*-PGP-9 substrates. This substrate range could facilitate cross-resistance, which indeed has been reported in *Parascaris* sp. [47].

In *C. elegans*, only one study had previously demonstrated the impact of a transgenic Pgp on IVM susceptibility, by overexpressing *Pun*-PGP-11.1 under the control of the native *Cel-pgp-11* promotor [41], which has been shown to primarily drive intestinal expression [48]. The concordantly observed moderate fold changes in this and Janssen et al., despite the extrachromosomal array overexpression, might indicate that Pgps cannot cause resistance by themselves in parasitic nematodes but rather that Pgps are contributors within a multigenic context as proposed by NGS studies in other nematode species [17,18].

### 3.2. The Intestinal and Transcuticlular-Epidermal Uptake of Ivermectin and Moxidectin

Interestingly, the extent of the protective effect against IVM resulting from the *Pun*-PGP-9 expression at the intestine or the epidermis was markedly influenced by the selective intestinal exposure from active drug ingestion by PP. By itself, this newly described effect of active drug ingestion on ML susceptibility was considerable and, for IVM, even surpassed the extent of the effect of Pgp overexpression in either tissue.

The differences between the effect on MOX and IVM susceptibility of active drug ingestion and intestinal or epidermal *Pun*-PGP-9 overexpression might be interpreted as differing uptake capacities at specific *C. elegans* tissues. As a general mechanism, PP stimulation is required for food uptake, and the increased uptake of incubation fluid also facilitates the accumulation of its contents [36]. 

For IVM, the strong effect of active drug ingestion and the PP dependency of the protective effect of intestinal *Pun*-PGP-9 expression indicate that higher intestinal exposure increases the overall concentration and effect of IVM. Nonetheless, the measurable impact of epidermal *Pun*-PGP-9 expression on susceptibility also indicates that IVM is taken up, although less efficiently, via the cuticle–epidermis.

In contrast, the effect of epidermal *Pun*-PGP-9 expression on MOX susceptibility regardless of PP could suggest that the epidermis–cuticle is the predominant MOX uptake route. For IVM and a few other anthelmintics, transcuticular-epidermal permeability had already been described by biophysical studies on cuticle–epidermis preparations of *Ascaris suum* and experiments using live *A. suum* with a surgically ligated pharynx [49,50]. Likewise, experiments in *C. elegans* have demonstrated a considerably quicker onset of pharyngeal paralysis in a cuticle defective *C. elegans bus-8* loss-of-function strain [51]. A study by O’Lone and Campbell indicated that PP inhibition by refrigeration, and thus inhibition of oral ingestion, reduced the susceptibility to IVM in *C. elegans* [52]. The authors suggested that oral ingestion increases IVM susceptibility but that transcuticular-epidermal uptake is sufficient to induce paralysis at comparatively low concentrations [52], which is in high concordance with our observations and conclusions.

In contrast to IVM, the uptake of MOX in nematodes has not been studied at all. The absence of a similarly strong effect of PP on MOX susceptibility as observed for IVM could indicate that the cuticle–epidermis represents the main MOX penetration route. This conclusion is also supported by the lowest MOX susceptibility regardless of PP stimulation in the epidermis-Pgp-9 strain. The main factor determining permeability through the nematode cuticle is compound size [49,50,53], and MOX is considerably smaller than IVM due to the lack of the C13 glycosyl side chain. Additionally, lipophilicity is an ancillary but important factor for transcuticular permeability [49,50,53], and indeed IVM has a lower lipophilicity than MOX [54]. These differences in size and lipophilicity seem to offer a reasonable explanation for a less efficient transcuticular IVM uptake but cannot explain why intestinal drug exposure from active drug ingestion increased only IVM but not MOX susceptibility. Therefore, more detailed studies are needed to understand this difference. Both ML derivatives are very lipophilic and accumulate well in high-fat-content tissues in the host [55]. In this regard, the mainly intestinal and epidermal fat storage in *C. elegans* [56] should facilitate passive diffusion and accumulation in these tissues.

### 3.3. Experimental Limitations and Relevance for Parasitic Nematodes

The novel insights from the present study are, to some extent, limited to the model nematode *C. elegans* that was utilised, since the required forward genetic tools are not yet available for *Parascaris* sp. or any related parasitic nematode species [57]. However, it may be anticipated that the general conclusions of the tissue-specific protective function in *C. elegans* may be extrapolated as a general functional mechanism of Pgps in nematode barrier tissues, such as the epidermis and the intestine, in light of a common nematode body structure. Nevertheless, it should be noted that parasitic nematodes exhibit differences in size, life cycle, stage-specific feeding behaviour, genetics, e.g., in their receptor repertoire [58] and, hence, in their drug susceptibility [59]. For example, IVM and MOX EC_50_ values for adult *C. elegans* were several fold higher than those measured in the adult stage of examined parasitic nematodes in vitro, e.g., *T. circumcincta* [60]. The reasons for these differences remain elusive and have been a matter of speculation. In non-feeding third larval stages, exposure to MLs (or any other anthelmintic) is restricted to the cuticle and, indeed, non-feeding larval stages of parasitic nematodes exhibit considerably lower susceptibilities [60,61]. These differences might be explained by an increased susceptibility from active IVM ingestion. Furthermore, the sheath (i.e., the residual cuticle of the second larval stage) of the third-stage strongylid larva might also reduce susceptibility by limiting drug penetration into the worm. Regarding the uptake of MLs in adult stages of parasitic worms, an in vivo study using an ML-resistant population of the sheep parasite *H. contortus* found no difference of initial drug accumulation in parasites between MOX, IVM, and abamectin 0.5 days after treatment [62]. However, MOX concentrations in the host dropped significantly compared to IVM and abamectin despite similar plasma levels within 2 days post treatment, suggesting that drug elimination may vary between different ML derivatives [62]. In *Ostertagia ostertagi*, a cattle parasite dwelling on the surface of the abomasal mucosa and feeding by sucking on the mucosa, IVM levels in the worms were shown to correlate with IVM concentration in the abomasal mucosa and were maximised by subcutaneous drug application [63]. These findings would support that parasite nematode IVM susceptibility is also increased by feeding. In the same study, IVM levels in the cattle parasite, *Cooperia oncophora*, which dwells in the small intestine, correlated well with intestinal content, and parasite exposure was maximised by oral drug application [63]. Overall, the study by Leathwick et al. highlights how parasite exposure considerably differs between parasitic species with regard to their biology [63], a concept that can also be extended to the different developmental stages, e.g., migrating/histotropic or hypobiotic stages. Based on the feeding behaviour, the blood-sucking *H. contortus* would be expected to experience high intestinal ML exposure during the early peak in plasma concentrations and, thereafter, prolonged low intestinal exposure, while the parasite cuticle would be exposed from the drug in the abomasal fluid [62,64,65]. Measuring IVM concentrations in adult *H. contortus* 3 days after subcutaneous injection, Lloberas et al. reported only a weak correlation with plasma levels, possibly because of the timing of the measurement [64]. In contrast, intraruminal IVM application resulted in very high abomasal drug concentration, which also resulted in high concentration in the parasite tissues 3 days after IVM application, suggesting an efficient transcuticular uptake [64].

Regarding the observed tissue-specific expression patterns, some reservations remain whether expression was exclusive to the targeted tissues, the intestine and the epidermis. The representative images in Figure 1 show the principal expression pattern differences observed in the stained worms between the lines with transgenic expression induced by two well-characterised promotors, ges-1p and col-19p. However, extrachromosomal array overexpression might result in background expression in other tissues and in particular epidermal specificity was difficult to verify as fluorescence was visible throughout the worm and staining appeared variable along the length of all stained worms. The fluorescence visible in the entire worm might be explained by autofluorescence as it appeared in both the green and blue channels (Figure 1c). The variability in the strength of the staining was also observed in other worms and could be the result of a non-uniform antibody exposure and deformation resulting from the patchy freeze-cracking. 3D-images would be better suited to verify tissue specificity and to rule out background expression in unwanted tissues [66].

## 4. Conclusions

**Figure 5:**
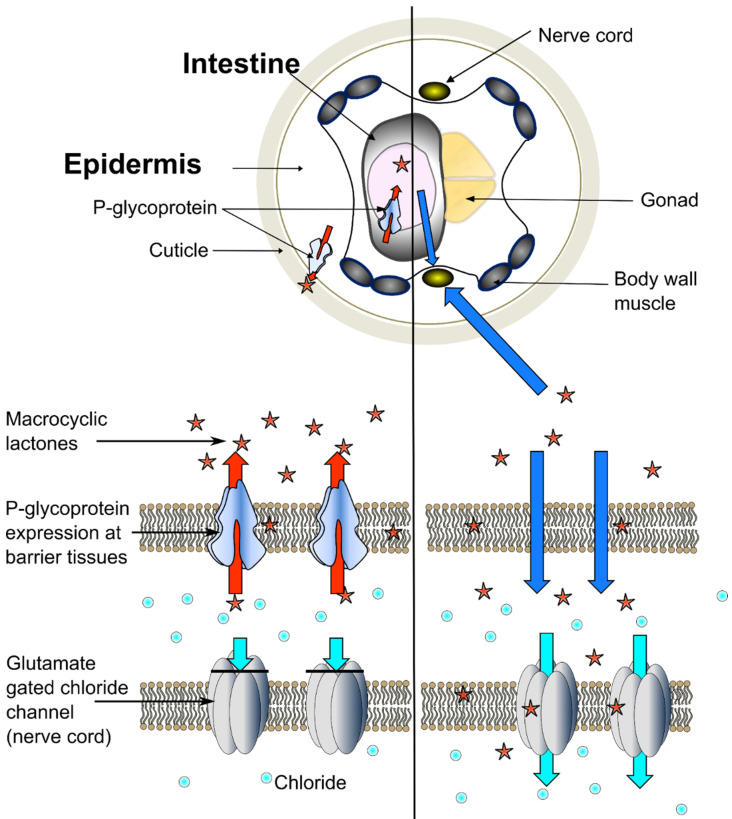
Schematic illustration of P-glycoprotein-mediated barrier function. Hypothetical schematic illustration of Pgp-mediated barrier function in a *Caenorhabditis elegans* adult. Expression of P-glycoproteins in specific barrier tissues, i.e., the epidermis and the intestine prohibit MLs from reaching target tissues, thereby preventing an ML-induced hyperpolarisation of the neurons and muscle paralysis.

Our results demonstrate that Pgps can contribute to reducing ML susceptibility in a tissue-specific manner in nematodes and that active drug ingestion increases ML susceptibility. The observed dependency of the protective effect of intestinal or epidermal Pgp overexpression on active drug ingestion would suggest a role of Pgps in barrier fortification by reducing tissue drug permeability as a possible mechanism of a Pgp-mediated ML susceptibility as illustrated in Figure 5. As the tissue-specific expression patterns vary considerably between the diverse Pgp lineages in different nematode species [23,27], the protective capacity of a specific Pgp lineage will vary between species and developmental stages.

Furthermore, our findings emphasise that more attention should be placed on how target parasite species take up ML drugs, since this might lead to differences in susceptibility to individual ML derivatives. Whether changes in barrier permeability represent a relevant mechanism of anthelmintic resistance remains to be elucidated in parasitic nematodes.

In conclusion, this study significantly improves the understanding of a Pgp-mediated ML resistance mechanism by demonstrating how transgenic Pgp expression at specific barriers can impact the susceptibility to different ML derivatives. Furthermore, the differing relevance of active drug ingestion for IVM and MOX susceptibility suggests thus far unknown pharmacological differences and demonstrates the importance of drug barriers and uptake routes for susceptibility.

## 5. Materials and Methods

### 5.1. Plasmids and Plasmid Construction

Plasmids for transgenesis were assembled using the NEB HIFI DNA Assembly Kit (New England Biolabs Inc., Ipswich, MA, USA) according to the manufacturer’s instructions into the pUC19 vector linearised with *Sma*I (ThermoFisher, Waltham, MA, USA). Plasmid constructs were *Cel-col-19p::Pun-pgp-9::FLAG::Cel-unc-54_3′-UTR* utilising the *col-19* promotor [39] to drive epidermal *Pun*-PGP-9 expression (Supplementary Figure S3a) and *Cel-ges-1p::Pun-pgp-9::FLAG::Cel-unc-54_3′-UTR* (Supplementary Figure S3b) utilising the *ges-1* promotor [38] to drive intestine-specific *Pun*-PGP-9 expression. The *C. elegans unc-54* 3′-UTR [41] and the *Pun*-pgp-9 cDNA [23] were amplified from verified plasmids, while the 3′ end primer for the *Pun-pgp-9* amplification introduced an in-frame FLAG-tag (DYKDDDDK) before the stop codon (all primers in Supplementary Table S3). The *C. elegans* promotors *col-19p* and *ges-1p* [38] were amplified from genomic DNA extracted from the Bristol N2 strain [39]. A co-injection marker plasmid (pPD118.33) driving pharyngeal GFP expression was used (Addgene L3790 plasmid #1596 was a gift from A. Fire). Sequences of all constructs were confirmed by Sanger-sequencing (LGC Genomics, Hoddesdon, UK).

### 5.2. Generation of Caenorhabditis elegans Strains and Maintenance

*Caenorhabditis elegans* WT strain Bristol N2 was obtained from the *Caenorhabditis* Genetics Centre (CGC; University of Minnesota, Minneapolis, MN, USA) and the *Cel-pgp-9* deletion strain tm830 [*Cel-pgp-9(-)*] was obtained from the National BioResource Project (NBRP; Tokyo, Japan). Strains were maintained at 20 °C and standard conditions on NGM plates [67].

Plasmid constructs for intestinal and epidermal *Pun*-PGP-9 expression were loaded onto Eppendorf FemtoTips II needles at 25.0 ng/µL along with pPD118.33 as a co-injection marker at 12.5 ng/µL. Injection into the gonads of 1-day adults of the *Cel-pgp-9* deletion strain was performed using an Eppendorf Femtojet 4 connected to an Eppendorf micromanipulator and mounted onto a Leica inverse microscope. Preparation of worms for injection was carried out as described elsewhere [68]. Additionally, a strain controlling for pharyngeal GFP expression and presence of extrachromosomal arrays in general was generated by injecting only the co-injection marker. Transgenic strains carrying extrachromosomal arrays *IntPgp-9Ex1* and *IntPgp-9Ex2* with genotype [*Cel-pgp-9(-); Cel-ges-1p::Pun-pgp-9::FLAG::Cel-unc-54_3′-UTR; Cel-myo-2p::gfp::Cel-unc-54_3’UTR], EpiPgp-9Ex1* with genotype [*Cel-pgp-9(-); Cel-col-19p::Pun-pgp-9::FLAG::Cel-unc-54_3′-UTR; Cel-myo-2p::gfp::Cel-unc-54_3′UTR]*) and *CtrlEx1* with genotype [*Cel-pgp-9(-); Cel-myo-2p::gfp::Cel-unc-54_3′-UTR*) were maintained by regular transfer of GFP-positive individuals to a new plate.

### 5.3. Verification of Pun-PGP-9 Expression by RT-PCR and Immunofluorescence

Transcription of *Pun-pgp-9* in GFP-positive offspring of injected worms was confirmed by reverse transcriptase (RT)-PCR (primers and cycle conditions in Supplementary Table S3) using the S7 Fusion enzyme (Mobidiag) and a cDNA or a no-RT control template. cDNA was generated from DNAse-treated RNA. In addition, tissue-specific *Pun*-PGP-9 protein expression was examined with immunofluorescence. Freeze cracking and antibody staining were performed in a 1.5 mL tube as described elsewhere [69] with minor adaptations using 4% formaldehyde and 50% methanol for fixation followed by freeze-cracking worms three times in liquid nitrogen and thawing in water at room temperature. Following the freeze–thaw cycles, tubes were shaken at 37 °C for 1 h. To remove the fixative, worms were centrifuged at 11,000× *g* for 1 min and washed with PBS-T (phosphate-buffered saline +0.5% Triton-X100) four times, removing all liquid during the last aspiration of PBS-T. Prior to antibody staining, worms were incubated in 1 mL PBS-BSA (PBS + 1% bovine serum albumin) overnight at 4 °C under mild shaking. The next day, worms were centrifuged for 1 min and then incubated for 24 h at 4 °C and mild shaking in 500 µL PBS-BSA containing a monoclonal (FG4R), mouse-derived anti-FLAG IgG antibody diluted 1:200 (ab125243, A85282 antibodies.com). The following day, worms were washed again five times in PBS to remove unbound primary antibody. Once again, worms were incubated for 24 h at 4 °C in 500 µL of PBS-BSA containing DyLight 405 conjugated polyclonal donkey anti-mouse antibodies diluted 1:300 (DyLight™ 405 AffiniPure Donkey Anti-Mouse IgG (H+L), Jackson ImmunoResearch). Following another five washes with PBS-BSA, all liquid was completely removed, and worms were transferred in ~25 µL to an untreated microscope slide. After adding a drop (~25 µL) of VECTASHIELD^®^ mounting medium and sealing with nail polish, specimens were examined on an Eclipse Ti-U inverted research confocal microscope (Nikon, Tokyo, Japan) with a 20× and a 40× objective at excitation wavelength 405 nm to visualise antibody-specific staining (DyLight405) and 488 nm excitation to visualise pharyngeal GFP expression. Differential interference contrast (DIC) pictures were taken to visualise worm anatomy. Images were taken using VisiView 4.3.0 at 16 bit. ImageJ was used to pseudocolour and merge channels [40]. Fluorescent images at 405 nM and 488 nm excitation wavelength were visualised in the blue or green lookup table (LUT) scale provided by ImageJ.

### 5.4. Trashing Assay

To prepare stock solutions, IVM and MOX (Sigma-Aldrich, St. Louis, MI, USA) were dissolved in DMSO and frozen at −20 °C. A saturated 40 mM 5-HT stock solution was prepared by dissolving serotonin creatinine sulphate monohydrate (Sigma-Aldrich) in S-medium [67] through vigorous vortexing and was either immediately frozen at −20 °C or used directly.

Bleach-synchronised L1 [67] were grown to adult stage on NGM plates (72 h). One-day-old adults were washed with S-medium by repeatedly allowing worms to sink to the bottom of a 15 mL centrifuge tube, discarding, and refilling to remove all bacteria. The arrest of PP caused by the absence of food was confirmed visually after one hour of acclimatisation with an inverted microscope. In each individual experiment, 12 worms (only GFP-positive worms in case of transgenic strains) were transferred into 6-well plates (Sarstedt) with 2 mL S-medium final volume containing either no OP50 bacteria (OP50^−^), OP50 bacteria at OD_600_ = 0.5 (OP50^+^) or 4 mM 5-HT (OP50^−^/5-HT^+^ referred to as 5-HT^+^ in the text for simplicity), the latter two stimulating PP, which was visually confirmed before adding the drug. Dilution series of MLs were prepared in DMSO and added to the medium resulting in a final concentration of 1% DMSO. For IVM, the final concentrations for the OP50^−^ condition were 0.0, 10.0, 20.0, 30.0, 40.0, 50.0, and 100.0 nM IVM and for the OP50^+^ and 5-HT^+^ conditions 0.0, 1.0, 2.0, 3.0, 4.0, 5.0, and 10 nM IVM. For MOX, final concentrations for all conditions were 0.0, 5.0, 10.0, 12.5, 15.0, 17.5, 20.0, 30, 40, 50, and 100 nM MOX. Plates were sealed with parafilm to avoid evaporation and incubated in the dark at 20 °C and 150 rpm for 18–24 h. The next day, worms were transferred by pipetting to agar-coated Petri dishes filled with S-medium without bacteria and were allowed to acclimatise for 1 min. Then, thrashes of individual worms were counted for one minute on a stereo-fluorescence microscope.

*Caenorhabditis elegans* adult worms that were incubated in S-medium without bacteria did not readily move, and even soft touch stimulus and shaking did not induce any movements. However, transfer with a pipette induced strong thrashing, hence this step was performed across all experiments and all incubation conditions to detect ML-induced paralysis. Likewise, adult worms incubated with a sufficient supply of OP50 bacteria only thrashed occasionally, but the transfer of worms by pipetting induced vigorous thrashing.

### 5.5. Statistical Analysis

For each concentration, strain, and condition at least 12 worms per day were tested on three separate days (total *n* ≥ 36). Before log_10_ transformation of drug concentrations, vehicle controls were set to concentrations of 0.1 nM. Four-parameter non-linear logistic regression models and statistical analyses were calculated and visualised using GraphPad Prism 8.3.0 (GraphPad Software, San Diego, CA, USA) constraining the bottom values to ≥0. In each graph, the negative control concentration was visualised as “0 M no drug” with a break in the x-axis. Forest plots visualising corresponding half-maximal effective concentration (EC_50_) and 95% confidence intervals (CI) values were visualised using ggplot2 [42] in R v4.0.3 [43]. Statistical differences in EC_50_ values were calculated with the extra-sum-of-squares F test applying the Bonferroni–Holm correction for multiple testing and considering a p-value smaller than 0.05 as significant. For comparison, each *Pun*-PGP-9-expressing transgenic strain was compared to the control strain at the respective condition. Moreover, the control strain was compared to the WT at the respective condition. The EC_50_ values of the WT at the condition without food (OP50^−^) and without food plus 5-HT (5-HT^+^) were compared to the condition with food (OP50^+^). In addition, statistical comparisons of EC_50_ values between MOX and IVM were calculated for the WT. Fold changes were calculated based on EC_50_ values. To examine the impact of the feeding stimuli and transgene expression on motility, the motility response of all worms incubated without drug (negative no drug controls) were pooled per strain and per condition (*n* = 72) and a Kruskal–Wallis test with a Dunn’s post hoc was conducted comparing all conditions in each strain to the WT OP50^−^ condition in GraphPad.

## Data Availability

Plasmids are available upon request and will be deposited on Addgene.org. The data from this study are available on request from the corresponding author.

## Supplementary Materials

The following are available online at https://www.mdpi.com/1424-8247/14/2/153/s1, Figure S1: Concentration–response curves for intestine-Pgp-9 Line 2, wildtype, and control strain; Figure S2: Concentration–response curves of *Parascaris univalens* Pgp-9-expressing transgenic, control, and wildtype strains to ivermectin and moxidectin under different conditions; Figure S3: Vector maps of expression vectors, Table S1 Ivermectin concentration-response parameters of transgenic, control and wildtype strains at different conditions; Table S2 Moxidectin concentration-response parameters of transgenic, control and wildtype strains at different conditions; Table S3: Primer.
