# Supplementary material for: Pharyngeal Pumping and Tissue-Specific Transgenic P-Glycoprotein Expression Influence Macrocyclic Lactone Susceptibility in Caenorhabditis elegans
Source: Pharmaceuticals (Basel). 2021 Feb 13;14(2):153. doi: 10.3390/ph14020153 (PMC7917992; doi:10.3390/ph14020153)
Supplement: Supplementary file 1 [file pharmaceuticals-14-00153-s001.pdf]

Pharyngeal pumping and tissue-specific transgenic P-glycoprotein expression influence macrocyclic lactone susceptibility in *Caenorhabditis elegans*

Alexander P. Gerhard[1], Jürgen Krücken[1], Cedric Neveu[2], Claude L. Charvet[2], Abdallah Harmache[2], Georg von Samson-Himmelstjerna[1]

[1]Institute for Parasitology and Tropical Veterinary Medicine, Freie Universität Berlin, Germany
[2]INRAE, Université de Tours, ISP, F-37380, Nouzilly, France

* Correspondence:
samson.georg@fu-berlin.de

Supplementary Files

Supplementary Figure 1 Concentration-response curves for Intestine-Pgp-9 Line 2, wildtype and control strain
Supplementary Figure 2. All concentration-response curves of *Parascaris univalens* Pgp-9 expressing transgenic including both control and wildtype strains to ivermectin and moxidectin

Supplementary Figure 3 Vector maps of expression vectors

Supplementary Table 1 Ivermectin concentration-response parameters of transgenic, control and wildtype strains at different conditions

Supplementary Table 2 Moxidectin concentration-response parameters of transgenic, control and wildtype strains at different conditions

Supplementary Table 3 Primers

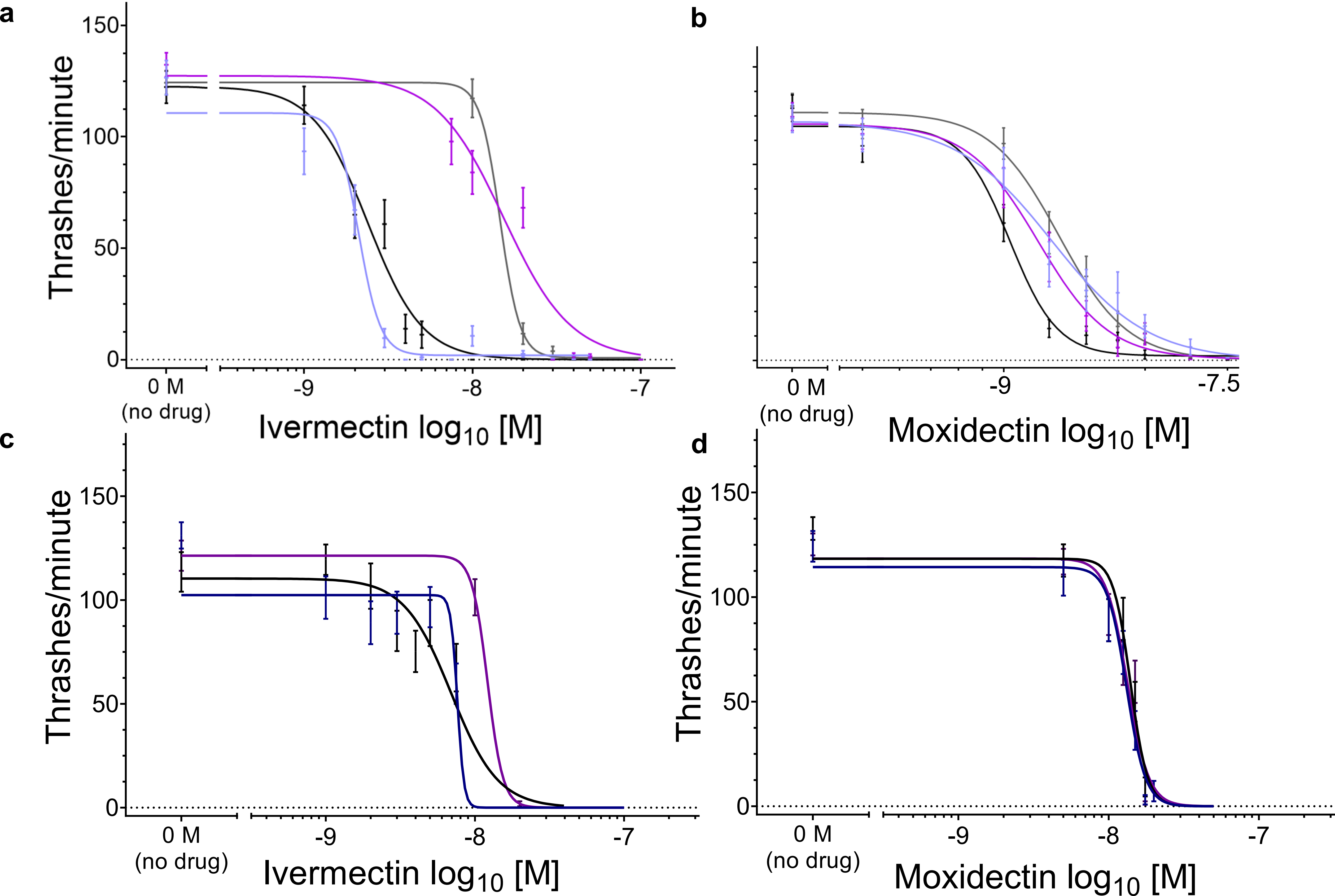

a
Thrashes/minute
150
100
50
0
0 M
(no drug)
-9
-8
-7
Ivermectin log10 [M]
b
0 M
(no drug)
-9
-7.5
Moxidectin log10 [M]
c
Thrashes/minute
150
100
50
0
0 M
(no drug)
-9
-8
-7
Ivermectin log10 [M]
d
Thrashes/minute
150
100
50
0
0 M
(no drug)
-9
-8
-7
Moxidectin log10 [M]

Supplementary Figure 1 Concentration-response curves for Intestine-Pgp-9 Line 2, wildtype and control strain

Concentration response curves were calculated and visualized in GraphPad v8.3.0 based on motility response (thrashes/minute) following ivermectin or moxidectin incubation. Concentrations were $\log_{10}$ transformed after setting the negative control to 0.1 nM. Concentrations response from four parameter non-linear regression models are visualized with the standard error of the mean at each concentration from 36 worms per concentration and condition. On the x-axis, the negative control was visualized as “0 M (no drug)” and separated by a break in the x-axis. Adult worms were incubated with a dilution series of ivermectin or moxidectin (final 1% DMSO concentration) for 24 hours under different conditions. **a-b** Motility response following **a** – ivermectin or **b** – moxidectin incubation in the wildtype (WT) and the control strain with pharyngeal pumping stimulation (PP) by 5-HT (black – control, blue WT) or without PP stimulation ($OP50^-$) (grey – control, purple – WT). **c-d** Motility response following **c** – ivermectin or **d** – moxidectin incubation in the intestine line 2 in the presence of PP stimulation by OP50 bacteria ($OP50^+$ - blue) and serotonin ($5\text{-}HT^+$ - black), or in the absence of a stimulus for PP ($OP50^-$ - purple). Pgp: P-glycoprotein; 5-HT is 5-hydroxytryptamine; N2Δ*Cel*Pgp-*9* is tm830 (NBRP); Transgenic strains genotypes: *EpiPgp-9Ex1* [*Cel-pgp-9(-); Cel-col-19p::Pun-pgp-9::FLAG::Cel-unc-54_3'-UTR; Cel-myo-2p::gfp:: Cel-unc-54_3’UTR*]; *IntPgp-9Ex1* and *IntPgp-9Ex2* [*Cel-pgp-9(-); Cel-ges-1p::Pun-pgp-9::FLAG::Celunc-54_3'-UTR; Cel-myo-2p::gfp::Cel-unc-54_3’UTR*]; *CtrlEx1* [*Cel-pgp-9(-); Cel-myo-2p::gfp::Cel-unc-54_3'-UTR*]

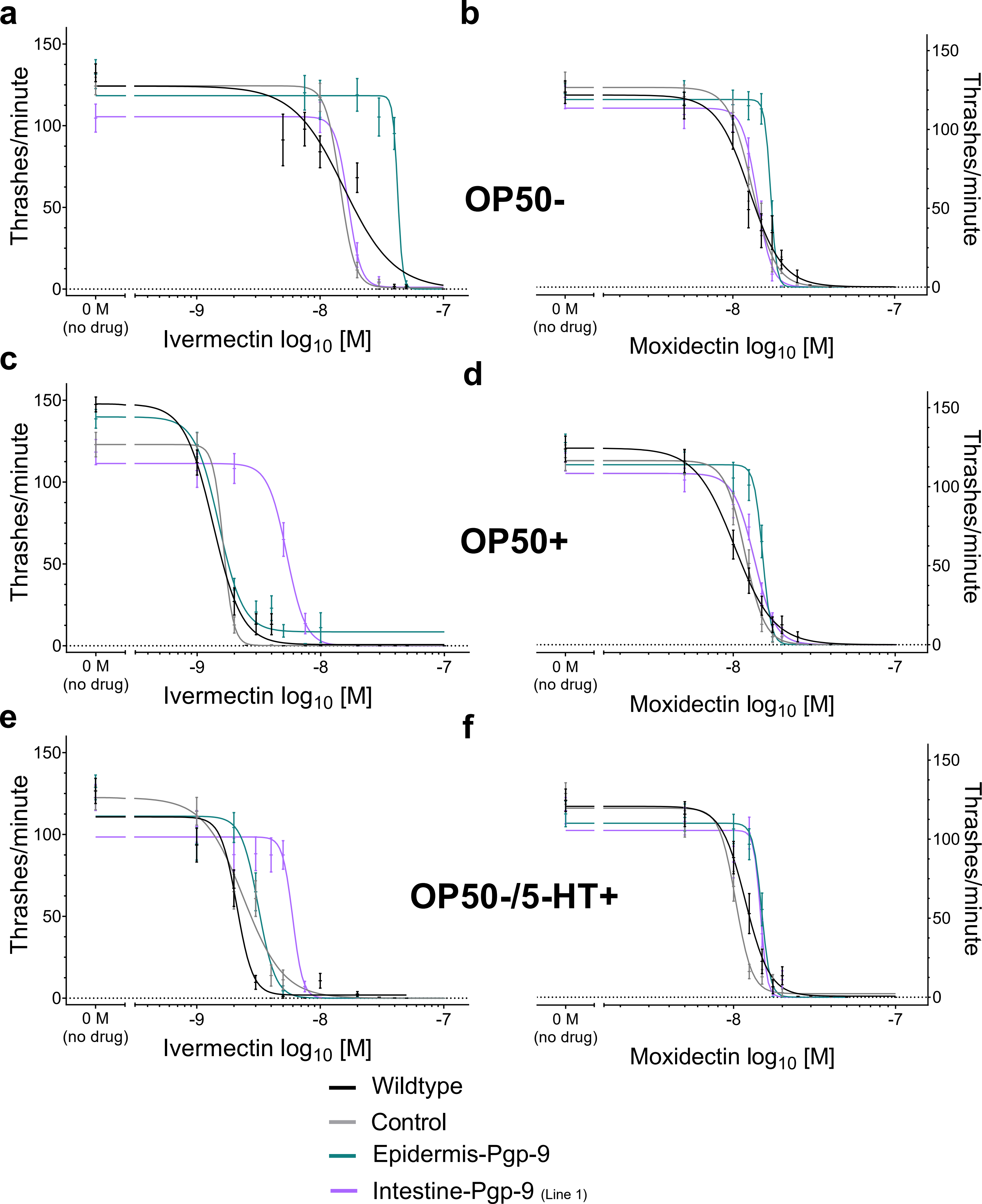
a
b
c
d
e
f
Thrashes/minute
Ivermectin log10 [M]
Moxidectin log10 [M]
0 M
(no drug)
OP50-
OP50+
OP50-/5-HT+
Wildtype
Control
Epidermis-Pgp-9
Intestine-Pgp-9 (Line 1)

Supplementary Figure 2. Concentration-response curves of *Parascaris univalens* Pgp-9 expressing transgenic, control and wildtype strains to ivermectin and moxidectin under different conditions

Concentration-response curves for ivermectin (a,c,e) and moxidectin (b, d, f) calculated with GraphPad v8.0.0 were visualized for 4 different strains, wildtype (WT) (black), control strain (grey), hypodermis-Pgp-9 strain (turquoise) and intestine-Pgp-9 line 1 (purple). For each strain and concentration, 36 synchronized 1-day old adult worms spread equally on three separate days were incubated for 24 hours in S-medium in the absence of OP50 bacteria ($OP50^-$) (**a**, **b**), in the presence of OP50 bacteria ($OP50^+$) (**c**, **d**) stimulating pharyngeal pumping, or in the absence of $OP50^-$ but with pharyngeal pumping stimulation by 4 mM 5-hydroxytryptamine ($OP50^-$/5-HT^+) (**e, f**). Four parameter logistic regression models were calculated and visualized along with the mean of trashes/minute (body bends) ± standard error of the mean at each concentration. Prior to the calculation, concentrations were $\log_{10}$ transformed and the no drug negative control was set to 0.1 nM. On the x-axis, the negative control was visualized as “0 M (no drug)” and separated by a break in the axis. For both drugs a dilution series with a final DMSO concentration of 1% was used. Pgp-9: P-glycoprotein-9

a

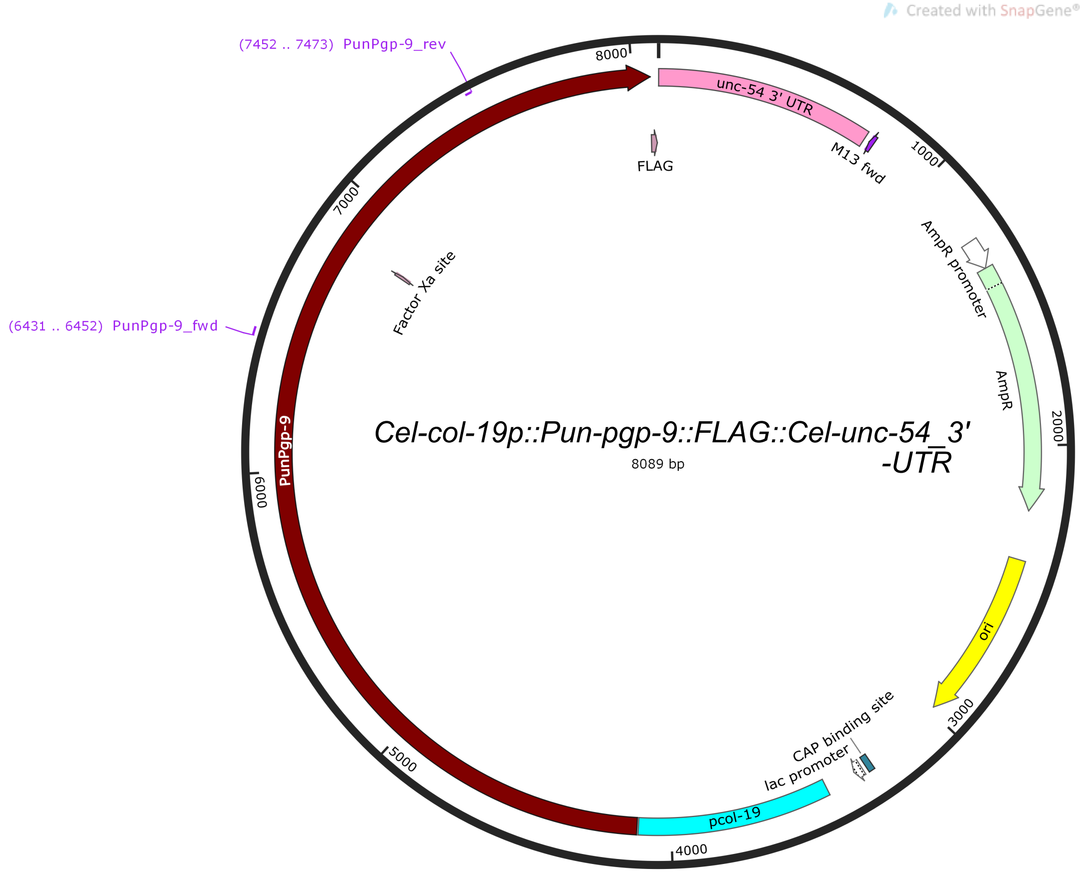

b

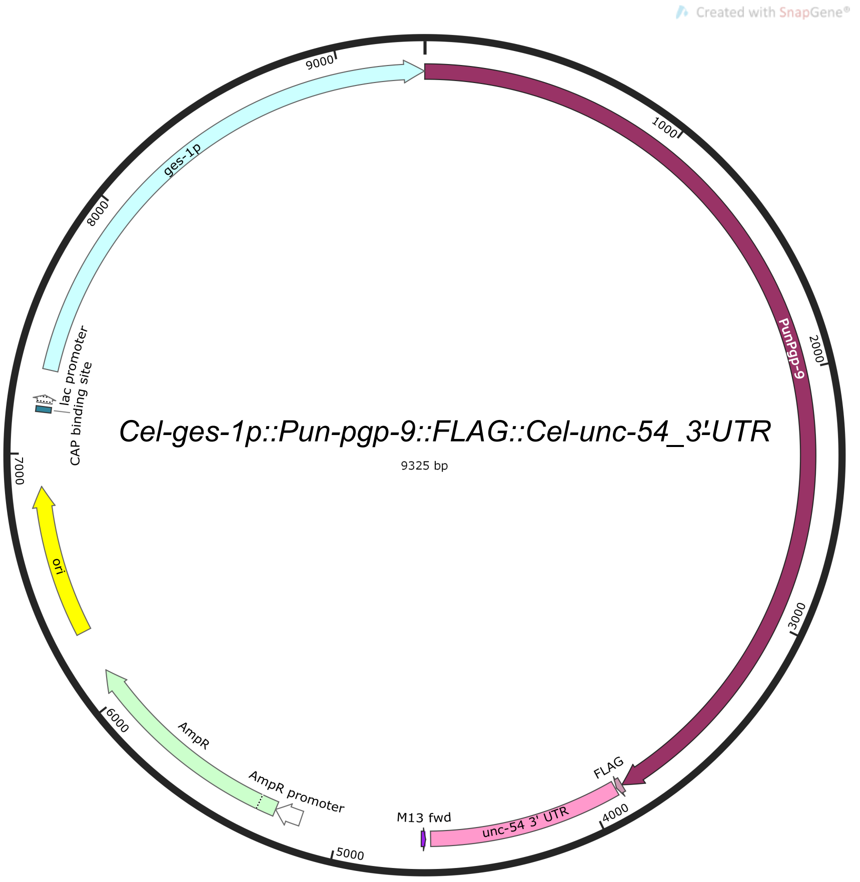

Supplementary Figure 3 Vector maps of expression vectors

Vector maps of plasmids driving tissue specific *Pun*Pgp-9 expression generated with SnapGene. The backbone vector puc-19 contains elements for transformation and selection in bacteria.

(**a**) *Cel*-col-19p::*Pun-pgp-9*::FLAG::*Cel-unc-54_3'-UTR* driving epidermal *Pun-pgp-9* expression

(**b**) *Cel-ges-1p*::*Pun-pgp-9*::FLAG::*Cel-unc-54_3'-UTR* driving intestinal *Pun-pgp-9*

Pun-pgp-9 is *Parascaris univalens* P-glycoprotein-9; UTR: untranslated region;

FLAG: FLAG-tag GAC TAC AAA GAC GAT GAC or DYKDDDDK (protein sequence)

Supplementary Table S1 Ivermectin concentration-response parameters of transgenic, control and wildtype strains at different conditions

Back-ground Strain	Strain (Genotype)[a]	***Pun*-PGP-9 expression**	**Condition**	**EC_{50}[b] (95% CI) [nM]**	**R^2**	**Fold Change[c]**	**p-value[d]**
N2	WT	–	$OP50^-$	16.68 (11.94-19.22)	0.58	11.1 (WT $OP50^+$)	0.0014
			$OP50^+$	1.50 (1.41-1.61)	0.78	–	
			$5\text{-}HT^+$	2.08 (1.97-2. 19)	0.61	1.4 (WT $OP50^+$)	0.0014
N2Δ*Cel-pgp*-9	Control	–	$OP50^-$	14.60 (12.79-16.67)	0.81	0.9 (WT $OP50^-$)	1
			$OP50^+$	1.51 (1.32-1.65)	0.79	1.0 (WT $OP50^+$)	1
			$5\text{-}HT^+$	2.37 (2.08-2.70)	0.60	1.1 (WT $5\text{-}HT^+$)	1
	Epidermis-Pgp-9 (*EpiPgp-9Ex1)*	Epidermis	$OP50^-$	42.62 (38.20-47.54)	0.46	2.9 (Control $OP50^-$)	0.0014
			$OP50^+$	1.53 (1.32-1.64)	0.69	1.0 (Control $OP50^+$)	1
			$5\text{-}HT^+$	3.16 (2.93 -3.41)	0.65	1.3 (Control $5\text{-}HT^+$)	0.0014
	Intestine-Pgp-9 Line 1 (*IntPgp-9Ex1)*	Intestine	$OP50^-$	18.29 (11.76-24.13)	0.63	1.2 (Control $OP50^-$)	1
			$OP50^+$	5.28 (4.81-5.84)	0.58	3.5 (Control $OP50^+$)	0.0014
			$5\text{-}HT^+$	6.00 (5.14-7.04)	0.41	2.5 (Control $5\text{-}HT^+$)	0.0014
	Intestine-Pgp-9 Line 2 (*IntPgp-9Ex2)*	Intestine	$OP50^-$	12.95 (9.73-15.15)	0.78	0.9 (Control $OP50^-$)	0.3102
			$OP50^+$	6.71 (4.77-9.44)	0.42	4.4 (Control $OP50^+$)	0.0014
			$5\text{-}HT^+$	7.63 (6.88-8.46)	0.59	3.2 (Control $5\text{-}HT^+$)	0.0014

Concentration-response parameters correspond to Figure 2C,E, Figure 3A-D and Figure 4.

[a]N2: N2 Bristol *C. elegans* strain; N2Δ*Cel*Pgp-9: *C. elegans* strain tm830; WT: wildtype (N2 Bristol *C. elegans* strain); Transgenic strains genotypes: *EpiPgp-9Ex1* [*Cel-pgp-9(-); Cel-col-19p::Pun-pgp-9::FLAG::Cel-unc-54_3'-UTR; Cel-myo-2p::gfp:: Cel-unc-54_3'UTR*]; *IntPgp-9Ex1* and *IntPgp-9Ex2* [*Cel-pgp-9(-); Cel-ges-1p::Pun-pgp-9::FLAG::Celunc-54_3'-UTR; Cel-myo-2p::gfp::Cel-unc-54_3'UTR*]; *CtrlEx1* [*Cel-pgp-9(-); Cel-myo-2p::gfp::Cel-unc-54_3'-UTR*]
[b]EC_{50}: half maximal effective concentration
[c]Fold changes were calculated by comparing the EC_{50} of a strain and condition to the EC_{50} of a respective control which is noted in brackets
[d]p-values were calculated by comparing a pair of non-linear regression models as listed in the fold-changes column using the extra-sum-of-squares-F test, and then adjusting p-values for multiple testing with the Bonferroni-Holm method in R
PGP/*pgp*: P-glycoprotein; CI: Confidence interval

Supplemetary Table S2. Moxidectin concentration-response parameters of transgenic, control and wildtype strains at different conditions

**Back-ground Strain**	**Strain (Genotype)**[a]	***Pun*-PGP-9 expression**	**Condition**	**EC_{50}[b] (95% CI) [nM]**	**R^2**	**Fold Change**[c]	**p-value**[d]
N2	WT	–	$OP50^{-}$	12.67 (11.60-13.84)	0.59	1.2 (WT $OP50^{+}$) and 0.76 (IVM WT $OP50^{-}$)	0.0024 and 0.1367
			$OP50^{+}$	10.19 (9.256-11.22)	0.63	6.7 (IVM WT $OP50^{+}$)	0.0003
			$5\text{-}HT^{+}$	11.85 (11.12-12.63)	0.65	1.2 (WT $OP50^{+}$) and 7.6 (IVM 5-HT^{+})	0.0196 and 0.0003
N2Δ*Cel-pgp*-9	Control	–	$OP50^{-}$	13.13 (12.44-13.87)	0.72	1.0 (WT $OP50^{-}$)	0.8892
			$OP50^{+}$	11.61 (11.24-12.40)	0.66	1.1 (WT $OP50^{+}$)	0.02
			$5\text{-}HT^{+}$	10.31 (9.93-10.72)	0.71	0.9 (WT 5-HT^{+})	0.0016
	Epidermis-Pgp-9 (*EpiPgp-9Ex1)*	Epidermis	$OP50^{-}$	17.15 (16.74-17.56)	0.69	1.3 (Control $OP50^{-}$)	0.0014
			$OP50^{+}$	15.18 (14.79-15.58)	0.69	1.3 (Control $OP50^{+}$)	0.0014
			$5\text{-}HT^{+}$	14.91 (14.48-15.35)	0.65	1.5 (Control 5-HT^{+})	0.0014
	Intestine-Pgp-9 Line 1 (*IntPgp-9Ex1)*	Intestine	$OP50^{-}$	13.31 (12.57-14.09)	0.65	1.0 (Control $OP50^{-}$)	0.1179
			$OP50^{+}$	13.96 (13.28-14.67)	0.65	1.2 (Control $OP50^{+}$)	0.0016
			$5\text{-}HT^{+}$	13.14 (12.39-13.93)	0.59	1.3 (Control 5-HT^{+})	0.0014
	Intestine-Pgp-9 Line 2 (*IntPgp-9Ex2)*	Intestine	$OP50^{-}$	14.13 (13.53-14.76)	0.62	1.1 (Control $OP50^{-}$)	0.8892
			$OP50^{+}$	13.49 (12.84-14.18)	0.58	1.2 (Control $OP50^{+}$)	0.0014
			$5\text{-}HT^{+}$	14.67 (14.19-15.16)	0.60	1.4 (Control 5-HT^{+})	0.0014

Concentration-response parameters correspond to Figure 2D, E, Figure 3A-D and Figure 4.

[a]N2: N2 Bristol *C. elegans* strain; N2Δ*Cel*Pgp-9: *C. elegans* strain tm830; WT: wildtype (N2 Bristol *C. elegans* strain); Transgenic strains genotypes: *EpiPgp-9Ex1* [*Cel-pgp-9(-); Cel-col-19p::Pun-pgp-9::FLAG::Cel-unc-54_3'-UTR; Cel-myo-2p::gfp:: Cel-unc-54_3'UTR*]; *IntPgp-9Ex1* and *IntPgp-9Ex2* [*Cel-pgp-9(-); Cel-ges-1p::Pun-pgp-9::FLAG::Celunc-54_3'-UTR; Cel-myo-2p::gfp::Cel-unc-54_3'UTR*]; *CtrlEx1* [*Cel-pgp-9(-); Cel-myo-2p::gfp:: Cel-unc-54_3'-UTR*]

[b]EC_{50}: half maximal effective concentration

[c]Fold changes were calculated by comparing the EC_{50} of a strain and condition to the EC_{50} of a respective control which is noted in brackets

[d]p-values were calculated by comparing a pair of non-linear regression models as listed in the fold-changes column using the extra-sum-of-squares-F test, and then adjusting p-values for multiple testing with the Bonferroni-Holm method in R

PGP/*pgp*:: P-glycoprotein; CI: Confidence interval

Supplementary Table 3 Primer

Name	Direction	Purpose	Sequence (5'-3')	Fragment Size (bp)*	Comment
Pun- Pgp-9_fwd	Forward	RT-PCR	GATCAGATGCTCAGCAATGGTC	1043 bp	
Pun- Pgp-9_rev	Reverse	RT-PCR	ACAGTTCCATCAATTGGGTCAT		
Cel- col-19p_fwd	Forward	Promotor amplification from gDNA	catttgaaaatttgcaccaatgt	671 bp	
Cel- col-19p_rev	Reverse		catcagttcatcaacatgcg		
ges-1p_puc19_ass_fwd	Forward	Assembly	gtcgactctagaggatccccaaactccgaactatgatg	2000 bp	Overlaps puc19 and *ges-1p*
ges-1p_puc19_ass_rev	Reverse	Assembly	taatagacatctgaattcaaagataagatatgtaatag		Overlaps *ges-1p* and *Pun-pgp-9*
col-19p_puc19_ass_fwd	Forward	Assembly	gtcgactctagaggatcccccatttgaaaatttgcaccaatg	671 bp	Overlaps puc19 and pcol-19
col-19p_puc19_ass_rev	Reverse	Assembly	taatagacatcgcatgttgatgaactgatg		Overlaps *col-19* p and Pun-pgp-9
Pgp-9_pcol-19_ass_fwd	Forward	Assembly	tcaacatgcgatgtctattagatcgagtcac		Overlaps *col-19p* and Pun-pgp-9
Pgp-9_pges-1_ass_fwd	Forward	Assembly	ttgaattcagatgtctattagatcgagtcac		
Pgp-9-FLAG_3UTR_ass_rev	Reverse	Assembly	tcacttatcatcatcatccttgtaatccatgcgtgcatcaaagtc	3972 bp	Overlaps unc-54 3'UTR and Pgp-9_5'-end and integrates a FLAG-tag, combined with a primer overlapping each promotor
unc-54-5'_Pgp-9-FLAG_ass_fwd	Forward	Assembly	gattacaaggatgatgatgataagtgaaggcccatctcgcgcccg	748 bp	Overlaps unc-54 3'UTR and Pgp-9_5'-end and integrates a FLAG-tag
unc-54-5'_ass_rev	Reverse	Assembly	tgaattcgagctcggtacccccctgcaggaaacagttatgtttggtatattgggaatgtattctg		Overlaps unc-54 3'UTR and puc19

*excluding flanking region of assembly primers

Supplementary Table S3 Primers
Primers for RT-PCR and assembly of plasmid constructs. Assembly primers were generated with NEBuilder Assembly tool (New England Biolabs). *Pun* : *Parascaris univalens* . *Cel* : *Caenorhabditis elegans* . Pgp: P-glycoprotein. UTR: untranslated region; gDNA genomic DNA
